# Clinical evaluation of palliative chemoradiotherapy for metastatic esophageal cancer

**DOI:** 10.18632/oncotarget.17925

**Published:** 2017-05-17

**Authors:** Hiroto Ueda, Masayuki Takeda, Shinya Ueda, Hisato Kawakami, Tatsuya Okuno, Naoki Takegawa, Hidetoshi Hayashi, Junji Tsurutani, Takao Tamura, Kazuki Ishikawa, Yasumasa Nishimura, Kazuhiko Nakagawa

**Affiliations:** ^1^ Department of Medical Oncology, Kindai University Faculty of Medicine, Osaka-Sayama, Osaka, Japan; ^2^ Department of Radiation Oncology, Kindai University Faculty of Medicine, Osaka-Sayama, Osaka, Japan

**Keywords:** concurrent chemoradiotherapy, advanced esophageal cancer, dysphagia, survival, platinum doublet

## Abstract

Platinum-based chemotherapy is considered a standard treatment option for patients with metastatic esophageal carcinoma. However, the overall survival of patients receiving such treatment is <1 year. A common presenting symptom of esophageal cancer is dysphagia, which has a substantial impact on quality of life. We have now retrospectively evaluated the efficacy and safety of palliative chemoradiotherapy for patients with stage IV esophageal cancer, most of whom are unfit for curative chemoradiotherapy. Fifty consecutive patients diagnosed with stage IV esophageal cancer were treated with concurrent chemoradiotherapy at Kindai University Hospital between April 2008 and December 2014. Most (90%) patients received a total radiation dose of at least 50 Gy, and the median number of treatment cycles per patient was four for the combination of 5-fluorouracil and cisplatin. The response of the primary tumor and the overall response were 80% and 44%, respectively. The dysphagia score was improved after chemoradiotherapy in 36 (72%) patients and did not change between before and after treatment in 14 (28%) patients. With a median follow-up time of 9.4 months from the start of chemoradiotherapy, the median progression-free survival and overall survival were 4.7 and 12.3 months, respectively. Three patients (T4b in two, T3 in one) developed esophagobronchial fistula after completion of chemoradiotherapy (*n* = 2) or after disease progression (*n* = 1), resulting in death in each case. Our results suggest that palliative chemoradioiotherapy was safe and contributed the improvement of dysphagia in patients with stage IV esophageal cancer.

## INTRODUCTION

Esophageal cancer is the eighth most frequently diagnosed cancer worldwide, and, despite improvements in surgical technique and the development of new approaches to treatment, it remains one of the most difficult malignancies to cure. About 50% of individuals with esophageal cancer already have metastatic disease at diagnosis and are therefore candidates for palliative therapy [1]. According to the National Comprehensive Cancer Network (NCCN) guidelines, chemotherapy with cisplatin and 5-fluorouracil (5-FU) is a standard treatment option for patients with metastatic esophageal carcinoma. However, the response rate to such treatment is only ∼35%, with an overall survival (OS) of <1 year [2–4].

Various factors influence a patient's decision to receive treatment for cancer. Local symptoms of esophageal cancer include dysphagia, odynophagia, cough, nausea, vomiting, regurgitation, and retrosternal pain. Dysphagia, or difficulty in swallowing food and liquids, is the most common and serious symptom of esophageal cancer and has a substantial impact on quality of life. In the event of persistent inadequacy of oral nutrient intake, patients with dysphagia require nutritional support, such as intravenous infusion or feeding via percutaneous gastrostomy or a nasogastric tube. For individuals with unresectable, metastatic esophageal cancer, long-term relief of dysphagia is one of the most important issues affecting daily life [5]. Many patients who present with symptoms of esophageal obstruction already have locally advanced or metastatic disease. Although intraluminal radiotherapy, intubation with self-expanding metal stents, and brachytherapy are effective palliative treatments to ameliorate dysphagia in patients with advanced esophageal cancer, the median survival of individuals receiving such noninvasive therapy is only ∼6 months [6, 7]. Chemotherapy alone requires several weeks to achieve symptom relief in such patients [8, 9]. Given that chemoradiotherapy with the combination of cisplatin and 5-FU is a standard therapy for patients with inoperable, locally advanced esophageal cancer and has a tolerable toxicity profile [10], the aim of the present study was to clarify the efficacy and safety of palliative concurrent chemoradiotherapy for patients with stage IV esophageal cancer who are unfit for curative chemoradiotherapy.

## RESULTS

### Patient characteristics

**Figure 1:**
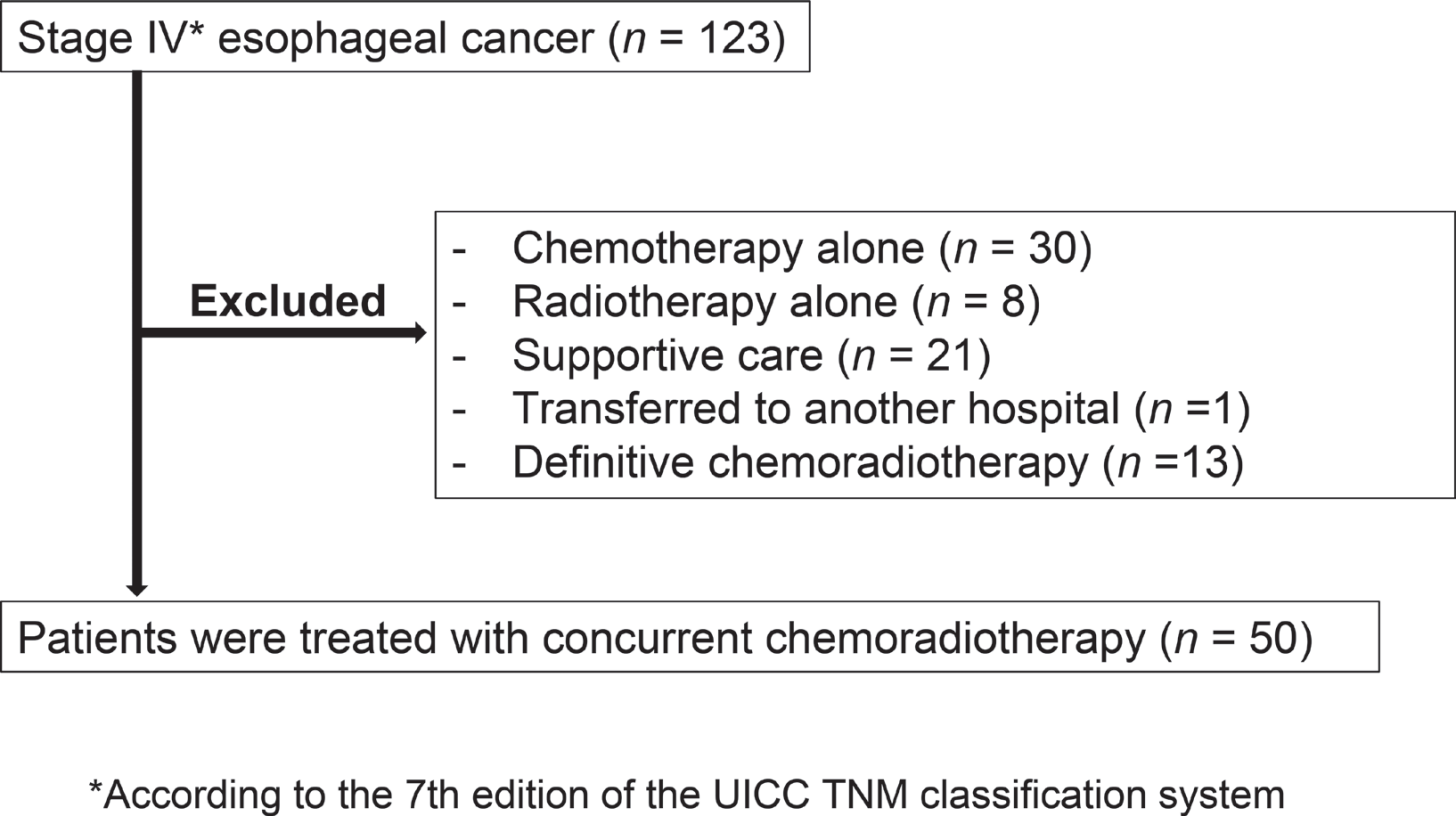
Patient flow diagram

Between April 2008 and December 2014, 123 consecutive patients were diagnosed with stage IV esophageal cancer according to the seventh edition of the Tumor-Node-Metastasis (TNM) classification system developed by the International Union Against Cancer (UICC) [11]. Seventy-three of these 123 patients were excluded from the study because they received chemotherapy (*n* = 30) or radiotherapy (*n* = 8) alone, they received definitive chemoradiotherapy in which supraclavicular or abdominal lymph nodes (M1 lymph nodes) were covered by the extended field of radiotherapy (*n* = 13), they received supportive care (*n* = 21), or they were transferred to another hospital (*n* = 1). The characteristics of the patients excluded from the study are shown in Supplementary Table 1. The proportion of patients with a good performance status was greater for those enrolled in the study compared with those who received supportive care, whereas the proportion of patients with severe dysphagia was greater for those enrolled in the study than for those who received chemotherapy alone. All excluded patients who underwent definitive chemoradiotherapy received 60 Gy of radiation. The 50 patients enrolled in the study were treated with concurrent chemoradiotherapy (Figure 1), with the demographics of these individuals being shown in Table 1. Forty-five (90%) patients were male and 47 (94%) were smokers, with the median age of all patients being 67 years (range, 44 to 78). Most (92%) patients had an Eastern Cooperative Oncology Group (ECOG) performance status of 0 or 1 at the time of testing. Forty-eight (96%) patients had squamous cell carcinoma. Twelve, 20, and 14 of the primary tumors were located in the upper, middle, and lower thoracic esophagus, respectively. Most (80%) patients had dysphagia of grade ≥ 2, with only one (2%) patient being free of dysphagia. Forty-three (86%) of the study subjects received 5-FU and cisplatin with concurrent radiotherapy, six (12%) received 5-FU and nedaplatin, and one (2%) received 5-FU alone. The decision to treat the latter patient with 5-FU alone was based on his advanced age (78 years) and the possibility of renal vein thrombosis (as revealed by contrast-enhanced computed tomography) that might have increased the risk for vascular complications of platinum chemotherapy.

**Table 1:** Characteristics of the study patients (*n* = 50)

	Subset	No. of patients (%)
[Median (range) age in years		67 (44–78)]
Sex	Male	45 (90)
	Female	5 (10)
Smoking status	Never	3 (6)
	Smoker	47 (94)
ECOG performance status	0–1	46 (92)
	2–3	4 (8)
Tumor histology	Squamous cell carcinoma	48 (96)
	Adenocarcinoma	2 (4)
Tumor location	Cervical	3 (6)
	Upper thoracic	12 (24)
	Middle thoracic	20 (40)
	Lower thoracic	14 (28)
	Abdominal	1 (2)
T stage	T1	0 (0)
	T2	1 (2)
	T3	32 (64)
	T4a/T4b	3 (6)/14 (28)
N stage	N0	1 (2)
	N1	28 (56)
	N2	8 (16)
	N3	13 (26)
Metastasis sites	Lymph nodes	40
	Liver	19
	Lung	19
	Bone	13
	Adrenal gland	3
	Kidney	1
	Peritoneum	1
	Pleura	2
[Median (range) tumor length (cm)		6 (3–20)]
Dysphagia score	0 (asymptomatic)	1 (2)
	1 (eat solid diet with some dysphagia)	9 (18)
	2 (eat semisolid diet)	28 (56)
	3 (drink liquid diet)	8 (16)
	4 (complete dysphagia)	4 (8)
Chemotherapy regimen	5-FU + cisplatin	43 (86)
	5-FU + nedaplatin	6 (12)
	5-FU alone	1 (2)
Total radiation dose (Gy)	54	2 (4)
	50	43 (86)
	< 50	5 (10)

### Treatment delivery and outcome

The median number of treatment cycles per patient was 4 for 5-FU and cisplatin (range, 1 to 6) and 3 for 5-FU and nedaplatin (range, 1 to 6). Forty-three (86%) of the 50 patients received a total radiation dose of 50 Gy, two (4%) patients received 54 Gy, and the remaining five (10%) patients received < 50 Gy (6, 11, 12, 20, or 44 Gy) (Table 1). The reasons for early withdrawal from radiotherapy included delirium (*n* =2), disease progression (*n* =1), cisplatin-induced acute renal failure (*n* =1), and withdrawal of agreement (*n* = 1). Eight (16%) patients treated with cisplatin plus 5-FU and three (6%) treated with nedaplatin plus 5-FU underwent a dose reduction for the second cycle as a result of adverse events.

**Figure 2:**
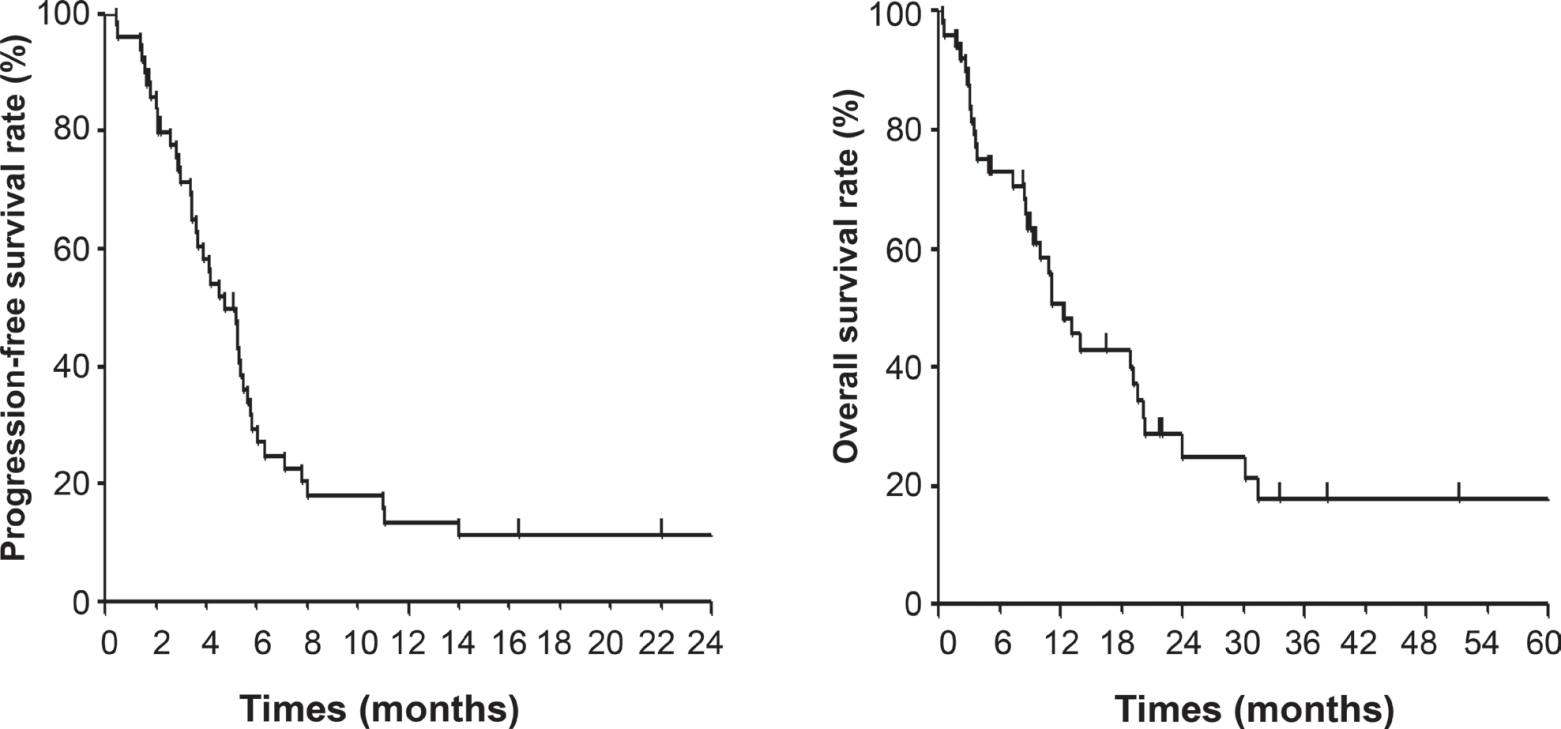
PFS (left) and OS (right) curves for patients diagnosed with stage IV esophageal cancer and treated with palliative chemoradiotherapy

The response of the primary tumor and the overall response are shown in Table 2. Among the primary tumors, five (10%) showed a complete response (CR) and 35 (70%) showed a partial response (PR), yielding a response rate of 80%. Four (8%) patients had stable disease (SD) and none (0%) had progressive disease (PD). With regard to the overall response, defined on the basis of the response at measurable primary and metastatic sites, one (2%) patient achieved a CR and 21 (42%) a PR. A total of 38 (76%) patients ultimately developed PD, with all of these individuals experiencing distant failure (defined as outside the radiation field). The most frequent sites of progression were the liver in 20 patients, lung in 15 patients, lymph nodes in 15 patients, and bone in nine patients. Among the 38 patients who ultimately developed PD, 17 individuals received treatment with docetaxel, two with paclitaxel, and one with S-1. After a median follow-up time of 9.4 months from the onset of chemoradiotherapy, 16 patients were still alive at last contact, with the median follow-up time for these individuals being 11.0 months. Kaplan-Meier curves for progression-free survival (PFS) and OS are shown in Figure 2. Median PFS and OS were 4.7 and 12.3 months, respectively (OS recalculated after extended follow-up in February 2017 is shown in Supplementary Figure 1).

**Table 2:** Tumor response in the study patients (n = 50)

	No. of patients (%)	
	Primary site	Overall
CR	5 (10)	1 (2)
PR	35 (70)	21 (42)
SD	4 (8)	11 (22)
PD	0 (0)	12 (24)
Not evaluable	6 (12)	5 (10)
Response (CR + PR)	40 (80)	22 (44)
Disease control (CR + PR + SD)	44 (88)	33 (66)

### Improvement of dysphagia

**Figure 3:**
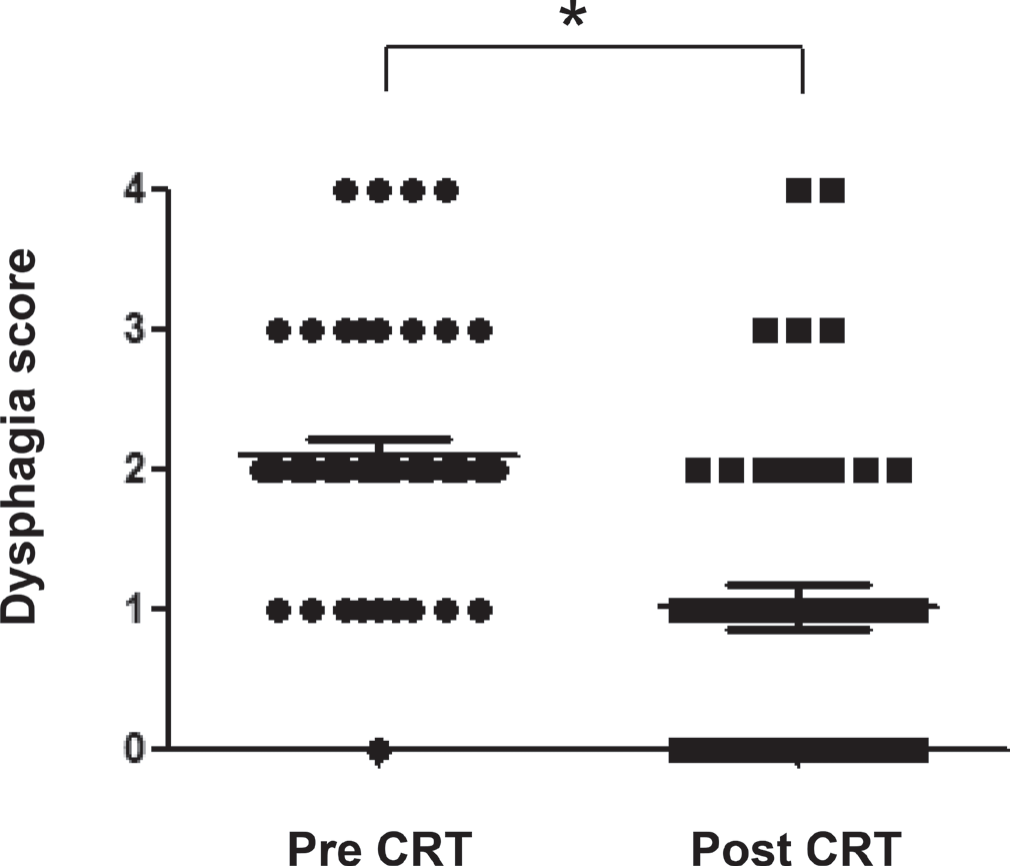
Distribution of dysphagia score (graded 0 to 4) before and after chemoradiotherapy (CRT) for the study patients The median scores are indicated by the horizontal lines. *P < 0.0001 (Mann-Whitney *U* test).

The distribution of dysphagia score (graded 0 to 4) for the study patients before and after chemoradiotherapy is shown in Figure 3. The dysphagia score had improved in most (72%) patients after treatment, with 19 individuals becoming dysphagia-free, whereas it remained unchanged in the remaining 14 (28%) patients. The median dysphagia score was 2 (able to swallow only semisolid foods) before treatment and 1 (able to swallow certain solid foods) after, with this change being statistically significant (*P* < 0.0001).

### Safety

The incidence of treatment-related adverse events is shown in Table 3. Hematologic toxicities of grade 3 or 4 included leukopenia (46%), neutropenia (46%), anemia (6%), thrombocytopenia (4%), and febrile neutropenia (10%), but all of these were manageable. The most common nonhematologic toxicities of grade 3 or 4 included anorexia (4%), hyponatremia (16%), and fatigue (4%). Other nonhematologic toxicities (all grades) related to radiation therapy included esophagitis in 31 patients, pneumonitis in eight patients, and pericardial effusion in six patients. Three (6%) patients (T4b in two and T3 in one) developed esophagobronchial fistula; this condition was apparent in two of these patients, who did not show disease progression, at 42 or 44 days after completion of chemoradiotherapy, whereas it was observed in the remaining patient at 68 days after detection of disease progression subsequent to completion of chemoradiotherapy. No patient died during chemoradiotherapy, although the three patients who developed esophagobronchial fistula died as a result of pneumonia. No other severe late life-threatening complications were observed in the present study.

**Table 3:** Adverse events occurring at any grade in the study subjects (n = 50)

	All grades, no. (%)	Grade ≥ 3, no. (%)
*Hematologic*		
Leukopenia	46 (92)	23 (46)
Neutropenia	39 (78)	23 (46)
Anemia	50 (100)	3 (6)
Thrombocytopenia	33 (66)	2 (4)
Febrile neutropenia	5 (10)	5 (10)
*Nonhematologic*		
Anorexia	26 (52)	2 (4)
Constipation	33 (66)	0 (0)
Mucosal inflammation	21 (42)	0 (0)
Esophagitis	31 (62)	0 (0)
Fatigue	24 (48)	2 (4)
Nausea	27 (54)	2 (4)
Vomiting	9 (18)	0 (0)
Diarrhea	5 (10)	0 (0)
Pyrexia	14 (28)	0 (0)
Elevated AST	12 (24)	0 (0)
Elevated ALT	18 (36)	0 (0)
Increased total bilirubin	9 (18)	0 (0)
Increased creatinine	17 (34)	2 (4)
Hyperkalemia	29 (58)	1 (2)
Hyponatremia	31 (62)	8 (16)
Pneumonitis	8 (16)	1 (2)
Esophageal perforation	3 (6)	3 (6)
Pericardial effusion	6 (12)	0 (0)

Abbreviations: AST, aspartate aminotransferase; ALT, alanine aminotransferase.

## DISCUSSION

Patients with inoperable metastatic esophageal cancer but with a good condition are candidates for palliative chemotherapy, such as that with the combination of cisplatin and 5-FU. However, the efficacy of such regimens has achieved a plateau, with the median OS being < 1 year [2]. New strategies are thus needed to maximize the efficacy of current treatments for such patients. We undertook the present investigation to assess the activity and tolerability of palliative concurrent chemoradiotherapy for patients with stage IV esophageal cancer, most of whom are unfit for curative treatment. The seventh edition of the TNM classification identifies supraclavicular lymph nodes and abdominal lymph nodes with the exception of celiac lymph nodes as sites of distant metastasis for esophageal cancer [11]. Thirteen patients were excluded from the present study because they received a curative extended field of radiotherapy that encompassed M1 (supraclavicular or abdominal) lymph nodes, with both prospective and retrospective studies having shown that patients with such lymph node metastasis achieve a better clinical outcome on chemoradiotherapy if these metastatic lymph nodes are covered by the extended radiotherapy field [12, 13]. Indeed, the excluded patients whose metastatic lymph nodes were covered by the extended field of radiotherapy showed a better clinical outcome than did the patients enrolled in our study (data not shown).

The overall response rate of 44% and OS of 12.3 months observed in the present study are similar to those obtained with concurrent chemoradiotherapy for locoregionally advanced esophageal carcinoma in the JCOG0303 trial (median OS of 13.1 months) [14], although this latter trial recruited a higher proportion of early-stage (curable) patients compared with our study, we excluded patients who received definitive chemoradiotherapy, and most of our patients experienced dysphagia, which is closely related to a poor outcome. A retrospective study of 40 patients with metastatic esophageal cancer treated with concurrent chemoradiotherapy consisting of 40 Gy of radiation plus the combination of 5-FU and cisplatin in the palliative setting reported an overall response rate of 55% and a median OS of 10.1 months [15]. The more favorable OS in our study (median of 12.3 months) might be due to the higher radiation dose administered or better control of the primary lesion.

We administered a higher radiation dose (50 Gy) than that (40 Gy) applied in the previous retrospective study [15]. The dose of radiation has been found to correlate significantly with the likelihood of achieving a tumor response in patients with esophageal cancer [16, 17]. Indeed, most (80%) patients in our study achieved a response for the primary tumor, which was accompanied by improvement in the dysphagia score in 72% of patients, with 19 individuals becoming dysphagia free. A reduction in tumor volume for the primary lesion during radiotherapy may also prolong the survival of patients with metastatic esophageal cancer. With regard to tumor histology, the incidence of adenocarcinoma of the esophagus has increased substantially in Western countries, whereas squamous cell carcinoma remains the major histological type of esophageal cancer in Japan and most other Asian countries [18]. Consistent with the notion that squamous cell carcinoma is more radiosensitive than is adenocarcinoma, the RTOG 85-01 study found that patients with squamous cell carcinoma of the esophagus treated with chemoradiotherapy tended to have a better clinical outcome than did those with adenocarcinoma [19]. A prospective trial is thus warranted to evaluate the effect of adding concurrent palliative radiotherapy to chemotherapy for patients with stage IV esophageal cancer.

Most (90%) patients in the present study completed chemoradiotherapy with a total radiation dose of at least 50 Gy, with only five patients discontinuing radiotherapy after a dose of < 50 Gy as a result of early disease progression, cisplatin-induced acute renal failure, withdrawal of agreement, or delirium, a neuropsychiatric complication in cancer patients. Leukopenia and neutropenia were the most common hematologic toxicities of grade 3 or 4, each being observed in about half of the study patients. The incidence of febrile neutropenia was only 10%. Esophagobronchial fistula is one of the most serious complications of advanced esophageal cancer [20, 21], and three (6%) patients in the present study developed this condition either after completion of chemoradiotherapy (*n* = 2) or after disease progression (*n* = 1), with all three patients dying from pneumonia. The death of the former two patients was considered treatment related, whereas that of the latter patient was not, given that a previous study found that fistula developed as a result of recurrent or persistent cancer after initial treatment of patients with esophageal cancer and that radiotherapy did not appear to be responsible [22]. The proportion of treatment-related deaths in the present study (4%) is similar to that in previous studies of chemoradiotherapy, which reported rates of 4% to 5% for patients with advanced esophageal cancer [15, 23] or 2.1% for those with locally advanced esophageal cancer [14]. Other adverse events observed in our study were manageable, suggesting that this therapeutic modality is tolerable and applicable to patients with advanced esophageal cancer in the palliative setting.

In conclusion, our retrospective study suggests that palliative chemoradioiotherapy was safe and contributed the improvement of dysphagia in patients with stage IV esophageal cancer who are unfit for curative chemoradiotherapy, although the possible development of fistula in a small number of patients is a potential concern. Given the inevitable biases potentially associated with such a retrospective study, this treatment approach warrants further prospective evaluation in larger populations.

## MATERIALS AND METHODS

### Patients

Consecutive patients with stage IV esophageal cancer who were treated with concurrent chemoradiotherapy at Kindai University Hospital between April 2008 and December 2014 were recruited to the study. Patients met all of the following criteria: (i) histologically confirmed esophageal cancer; (ii) clinical stage IV according to the seventh edition of the TNM classification system developed by the UICC; (iii) no previous chemotherapy; (iv) no previous thoracic radiotherapy; and (v) availability of clinical information during chemoradiotherapy. Patients with postresection recurrent esophageal cancer were excluded from the analysis, as were those who had supraclavicular or abdominal lymph nodes (M1 lymph nodes) covered by the extended field of radiotherapy. The choice of chemotherapy regimen was made by the treating physician. No restrictions on tumor histology, subsequent treatment, or performance status were imposed. Pretreatment staging evaluations included physical examination, laboratory tests, upper gastrointestinal endoscopy, and computed tomography (CT) scans from the neck to upper abdomen. Patient characteristics noted included sex, age, smoking history, ECOG performance status, tumor histology, tumor location, T stage, N stage, tumor length, metastatic sites, dysphagia score, chemotherapy regimen, and total radiation dose.

### Treatment

Chemotherapy consisted of 5-FU (700 or 800 mg/m^2^ on days 1 to 5, continuous) and either cisplatin (70 mg/m^2^ on day 1) or nedaplatin (80 or 90 mg/m^2^ on day 1) every 4 weeks for two cycles, with both cycles administered concurrently with radiotherapy. Cisplatin and nedaplatin were prepared in normal saline at the assigned dose and administered over 60 or 90 min, respectively, on day 1. After completion of the concurrent chemoradiotherapy, additional cycles of chemotherapy were administered to willing patients with sufficient bone marrow function and performance status. If a patient experienced excessive adverse events, a dose reduction was implemented for both drugs during the subsequent treatment cycle by the treating physician.

Radiotherapy was delivered with a linear accelerator featuring a 10-MV photon beam. A daily fractional dose of 2 Gy was administered for 5 days of each week up to a total dose of 50 Gy (25 fractions). The targeted area for esophageal carcinoma included the primary tumor with a 3.0-cm margin craniocaudally and nearby metastatic periesophageal lymph nodes with a 1.0-cm margin. Metastatic periesophageal lymph nodes in addition to the primary tumor were included in the radiation field to resolve dysphagia. After the delivery of 40 Gy, the radiation portals were reduced to shield the spinal cord and to encompass the gross tumor volume with a 1.0-cm margin, usually with the application of an oblique opposed field.

Dysphagia was measured before and immediately after completion of chemoradiotherapy. The dysphagia score was determined as previously described according to the following scale: 0, able to consume a normal diet (no dysphagia); 1, able to swallow certain solid foods; 2, able to swallow only semisolid foods; 3, able to swallow liquids only; and 4, unable to swallow anything (total dysphagia) [24]. All patients were hospitalized during chemoradiotherapy, thus allowing symptom evaluation and assessment of daily consumption of solid, semisolid, or liquid food.

### Evaluation of response and toxicity

Tumor response was examined by CT. Overall response was assessed according to the Response Evaluation Criteria in Solid Tumors (RECIST). For evaluation of the primary tumor, a PR was defined as a reduction of at least 30% in the volume of the primary tumor; PD was defined as an increase of at least 20% in the volume of the primary tumor; and a best response of SD required the criterion to be met for at least 6 weeks after initiation of treatment. A history was obtained and a physical examination, complete blood cell count, gastrointestinal endoscopy, chest x-ray, and CT scans of the neck, chest, and abdomen were performed about every 3 to 6 months after treatment initiation. Poststudy treatment was also at the discretion of treating physician.

Adverse events were recorded on the basis of the National Cancer Institute Common Toxicity Criteria (version 4.0). Toxicity was assessed during chemoradiotherapy and until disease progression. Treatment-related adverse events due to late toxicities such as esophageal fistula, radiation pneumonitis, other cardiopulmonary conditions, and radiation-induced gastric ulcer were also assessed after disease progression.

The primary end points of the study were the efficacy and tolerability of concurrent chemoradiotherapy for patients with stage IV esophageal cancer. Secondary end points were OS (time from initiation of treatment to the date of death from any cause or the date of last contact) and PFS (time from initiation of treatment to the date of progression, death without demonstrated disease progression, or last contact). The study was approved by the Institutional Review Board of Kindai University and conforms to the provisions of the Declaration of Helsinki.

### Statistical analysis

The probability of survival as a function of time was estimated with the Kaplan-Meier method. The difference in the distribution of dysphagia scores between before and after chemoradiotherapy was evaluated with the Mann-Whitney *U* test. A *P* value of < 0.05 was considered statistically significant. All statistical analysis was performed with GraphPad Prism software (version 5.0; GraphPad Software, San Diego, CA).

## SUPPLEMENTARY FIGURE AND TABLE


