# Supplementary material for: Clinical evaluation of palliative chemoradiotherapy for metastatic esophageal cancer
Source: Oncotarget. 2017 May 17;8(46):80286–94. doi: 10.18632/oncotarget.17925 (PMC5655197; doi:10.18632/oncotarget.17925)
Supplement: Supplementary file 1 [file oncotarget-08-80286-s001.pdf]

Clinical evaluation of palliative chemoradiotherapy for metastatic esophageal cancer

Supplementary Materials

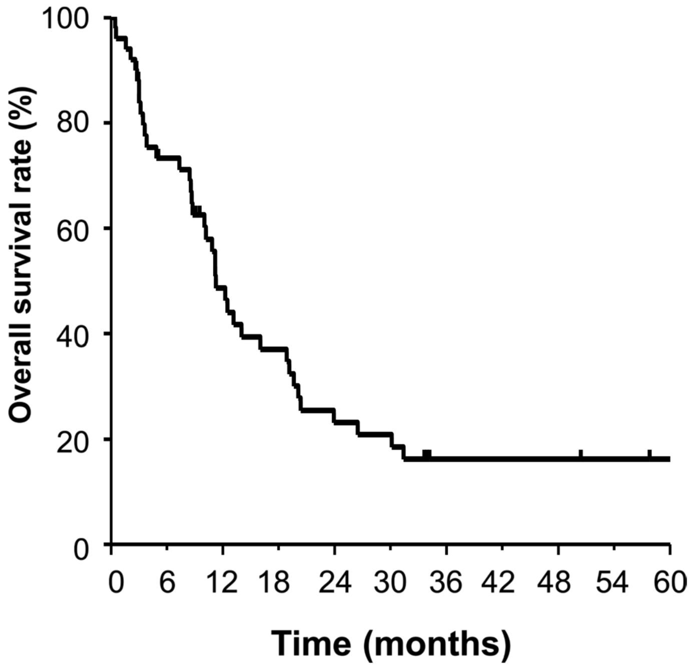

Supplementary Figure 1: OS curve for patients diagnosed with stage IV esophageal cancer and treated with palliative chemoradiotherapy. OS was recalculated after an extended follow-up period ending in February 2017 (median follow-up time of 10.4 months). Twelve patients, with a median follow-up time of 21.6 months, were still alive.

Supplementary Table 1: Characteristics of patients excluded from the analysis

	Subset	BSC (*n* = 21)	Radiotherapy (*n* = 8)	Chemotherapy (*n* = 30)	Definitive CRT (*n* = 13)
[Median (range) age in years		72 (46–83)	68 (63–81)	66 (43–76)	67 (43–75)]
Sex	Male	18 (86)	7 (88)	26 (87)	11 (85)
	Female	3 (14)	1 (13)	4 (13)	2 (15)
Smoking status	Never	2 (10)	1 (13)	3 (10)	0 (0)
	Smoker	12 (57)	7 (88)	25 (83)	13 (100)
	NE	7 (33)	0 (0)	2 (7)	0 (0)
ECOG performance status	0–1	8 (38)	5 (63)	29 (97)	13 (100)
	2–3	13 (62)	3 (38)	1 (3)	0 (0)
Tumor histology	Squamous cell carcinoma	18 (86)	7 (88)	26 (87)	12 (92)
	Adenocarcinoma	0 (0)	1 (13)	0 (0)	1 (8)
	Other	3 (14)	0 (0)	4 (13)	0 (0)
Tumor location	Cervical	3 (14)	1 (13)	1 (3)	0 (0)
	Upper thoracic	4 (19)	2 (25)	1 (3)	2 (15)
	Middle thoracic	8 (38)	3 (38)	15 (50)	9 (69)
	Lower thoracic	5 (24)	2 (25)	12 (40)	2 (15)
	Abdominal	1 (5)	0 (0)	1 (3)	0 (0)
T stage	T1	0 (0)	0 (0)	1 (3)	0 (0)
	T2	6 (29)	0 (0)	7 (23)	1 (8)
	T3	6 (29)	3 (38)	16 (53)	8 (62)
	T4a/T4b	2 (10)/7 (33)	2 (25)/3 (38)	1 (3)/5 (17)	0 (0)/4 (31)
N stage	N0	0 (0)	1 (13)	3 (10)	0 (0)
	N1	7 (33)	4 (50)	11 (37)	8 (62)
	N2	7 (33)	2 (25)	8 (27)	4 (31)
	N3	7 (33)	1 (13)	8 (27)	1 (8)
Metastasis sites	Lymph nodes	10	5	23	13
	Liver	7	1	11	0
	Lung	7	3	10	0
	Bone	3	0	5	0
	Adrenal gland	1	0	0	0
	Kidney	0	0	0	0
	Peritoneum	0	0	0	0
	Pleura	0	0	0	0
[Median (range) tumor length (cm)		7 (2–20)	6 (4–8)	6 (2–20)	8 (2–15)]
Dysphagia score	0	1 (5)	0 (0)	11 (37)	1 (8)
	1	2 (10)	2 (25)	11 (37)	1 (8)
	2	11 (52)	3 (37)	6 (20)	8 (62)
	3	2 (10)	2 (25)	2 (7)	3 (23)
	4	5 (24)	1 (13)	0 (0)	0 (0)
Chemotherapy regimen	5-FU + cisplatin			9 (30)	13 (100)
	5-FU + nedaplatin			5 (17)	0 (0)
	5-FU alone			0 (0)	0 (0)
	5-FU+docetaxel+nedaplatin			9 (30)	0 (0)
	5-FU+docetaxel+cisplatin			3 (10)	0 (0)
	Other			4 (13)	0 (0)
Total radiation dose (Gy)	62		1		0
	60		2		13
	54		1		0
	50		3		0
	< 50		1		0

Abbreviations: BSC, best supportive care; CRT, chemoradiotherapy; NE, not evaluable.
